# The Frog Skin-Derived Antimicrobial Peptide Suppresses Atherosclerosis by Modulating the KLF12/p300 Axis Through miR-590-5p

**DOI:** 10.3390/ijms262311497

**Published:** 2025-11-27

**Authors:** Fan Fan, Meng-Miao Li, Zhong-Peng Qiu, Zhen-Jia Li, De-Jing Shang

**Affiliations:** 1 School of Life Science, Liaoning Normal University, Dalian 116081, China; 2 Liaoning Provincial Key Laboratory of Biotechnology and Drug Discovery, School of Life Science, Liaoning Normal University, Dalian 116029, China

**Keywords:** antimicrobial peptide C-1b(3-13), atherosclerosis, miR-590-5p, KLF12, *ApoE*^−/−^ mice

## Abstract

Inflammation is a hallmark of atherosclerosis (AS), a complex chronic vascular disease. This study investigates the anti-atherosclerotic effects of the frog skin antimicrobial peptide(AMP) C-1b(3-13) in vitro and in vivo, focusing on the anti-inflammatory mechanism mediated by the miR-590-5p/KLF12/p300 axis in ox-LDL-induced PMA-THP-1 foam cells. MicroRNA(miRNA) sequencing was used to investigate the effects of AMP C-1b(3-13) on miRNA expression in ox-LDL-induced foam cells. Pro-inflammatory cytokine secretion regulated by miR-590-5p was detected by ELISA. Potential targets of miR-590-5p were bioinformatically predicted and validated through dual-luciferase reporter and RNA Immunoprecipitation(RIP)-qPCR assays. Western blot was used to assess the effects of C-1b(3-13) on Krüppel-like factor 12(KLF12), nuclear p300, and nuclear factor kappa B(NF-κB) pathway proteins; *ApoE*^−/−^ mice were utilized to establish the AS mouse model. Oil Red O (ORO) and hematoxylin and eosin (H&E) staining detected plaque formation and morphological changes in the aortic root. Immunohistochemistry analyzed CD68^+^(M1) and CD206^+^(M2) macrophage distribution within arterial plaques. miR-590-5p significantly suppressed pro-inflammatory cytokine secretion in ox-LDL-induced foam cells. Mechanistically, miR-590-5p directly targeted the 3′-untranslated region of *KLF12* mRNA, inhibiting KLF12 expression, reducing nuclear p300 accumulation, and subsequently attenuating NF-κB signaling pathway activation. Furthermore, AMP C-1b(3-13) treatment effectively attenuated inflammatory responses by upregulating miR-590-5p, which downregulated KLF12 expression, diminished nuclear p300 levels, and inhibited NF-κB signaling. In *ApoE*^−/−^ AS mice, C-1b(3-13) treatment markedly reduced aortic plaque formation, improved lipid metabolism, and suppressed inflammatory responses through the same signaling axis. These findings reveal a novel miR-590-5p-mediated regulatory mechanism in AS and identify AMP C-1b(3-13) as a promising therapeutic agent targeting miR-590-5p/KLF12/p300/NF-κB pathway.

## 1. Introduction

AS, a chronic inflammatory disease characterized by endothelial dysfunction, intimal lipid accumulation, smooth muscle cell proliferation, impaired cellular autophagy, and dysregulated apoptosis/necrosis, involves complex pathogenic mechanisms. Macrophages serve as pivotal cellular mediators in vascular intimal atherogenesis, with macrophage-derived foam cell formation representing a hallmark of early atherosclerotic lesions [1]. This process occurs when cholesterol influx and esterification exceed efflux capacity, leading to lipid-laden foam cell transformation. Lipid metabolism in macrophages encompasses cholesterol uptake, esterification/hydrolysis, and efflux pathways, all of which become dysregulated in AS [2]. The resultant lipid homeostasis disruption causes intracellular lipid overload and foam cell formation. These foam cells exhibit activated pro-inflammatory pathways, particularly the mitogen-activated protein kinase (MAPK) and NF-κB signaling cascades, which amplify inflammatory responses through enhanced cytokine secretion, thereby exacerbating plaque progression and AS development [3,4]. Consequently, therapeutic strategies targeting macrophage foam cell formation and pro-inflammatory activation represent crucial intervention points for AS management [5]. Over the past three decades, significant progress has been made in understanding atherogenesis, identifying cardiovascular risk factors, and developing statin-based therapies. Nevertheless, AS remains a leading global cause of mortality, underscoring the urgent need for novel therapeutic targets based on emerging cellular and molecular mechanisms [6,7].

Emerging evidence highlights microRNAs(miRNAs), a class of short (20–24 nucleotides) non-coding RNAs, as critical regulators in atherosclerotic pathogenesis [8]. Functioning through post-transcriptional gene silencing via mRNA degradation or translational inhibition, miRNAs are encoded in intergenic regions, introns, or clustered genomic loci [9]. These molecules regulate diverse biological processes, including cell growth, differentiation, migration, apoptosis, and angiogenesis. In AS pathology, miRNAs modulate monocyte/macrophage recruitment to plaque regions and regulate macrophage polarization toward pro-inflammatory or anti-inflammatory phenotypes. For instance, miR-27b reduces endothelial lipid uptake by targeting lipoprotein lipase (LPL) and fatty acid transport protein (FATP), while miR-148a modulates lipoprotein clearance through regulating LDL receptor expression [10,11]. In the context of inflammatory regulation, miR-21 drives macrophage M1 polarization via the PTEN/AKT/NF-κB pathway, and miR-125b participates in ox-LDL-induced inflammatory responses [12,13,14]. Notably, miR-181-3p exerts anti-inflammatory effects by blocking NF-κB signal transduction [15]. Collectively, these findings not only systematically elucidate the multidimensional regulatory mechanisms of miRNAs in AS but also provide a critical theoretical foundation for developing novel therapeutic targets.

AMPs, small bioactive polypeptides (12–50 residues) with amphipathic structures and cationic properties, constitute essential components of innate immunity. Over 3100 natural AMPs have been identified across species, exhibiting broad-spectrum antimicrobial activity against bacteria, fungi, and viruses [16,17]. Their eukaryotic cell compatibility, thermal stability, and low toxicity make them promising therapeutic candidates [18,19]. Particularly, AMP C-1b(3-13) represents a truncated analog of Chensinin-1b, which was designed based on the sequence of Chensinin-1 originally isolated from *Rana chensinensis* skin secretions. Structural optimization of Chensinin-1b involved strategic substitution of hydrophobic/polar residues, resulting in enhanced amphipathicity and bactericidal activity against both Gram-positive and Gram-negative bacteria. Further modification of the AMP C-1b(3-13) fragment (residues C-1b(3-13) of Chensinin-1b) through His-to-Arg substitutions at positions 4 and 10 generated derivative W3R6, which exhibits improved stability with a net charge of +6 [20]. Although our previous studies demonstrated that the frog skin-derived AMP C-1b and its analogs suppressed inflammatory responses, the underlying mechanism mediating their anti-inflammatory effects in atherosclerosis remains unclear [21,22]. MiRNAs have emerged as pivotal regulators in atherosclerotic inflammation. Through comprehensive miRNA profiling of ox-LDL-induced foam cells, we identified miR-590-5p as a dynamically regulated miRNA that responded to both ox-LDL stimuli and AMP C-1b(3-13)-treatment. Therefore, we proposed that miR-590-5p played key roles in foam cell formation. In this study, we elucidated the regulatory role of miR-590-5p in inflammatory responses in ox-LDL-induced foam cells, and investigated the anti-inflammatory mechanism of C-1b(3-13) via miR-590-5p signaling axis both in vitro and in vivo. This study not only identified a novel miRNA-mediated mechanism in AS but also positioned C-1b(3-13) as a promising candidate for AS therapy.

## 2. Results

### 2.1. Discovering Differentially Expressed miRNA Profiles by Sequencing in ox-LDL-Induced THP-1 Macrophages

**Figure 1:**
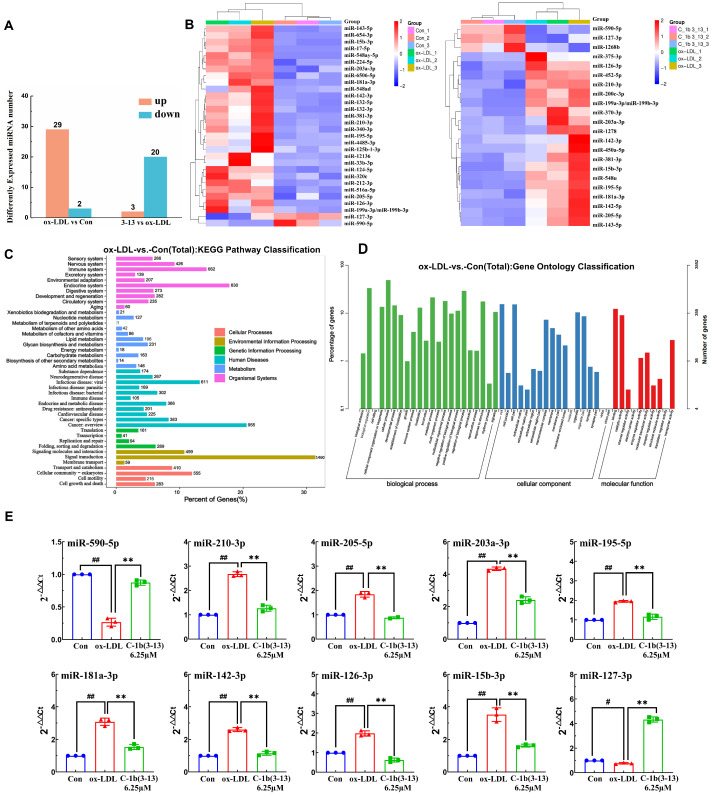
Differentially expressed miRNA profiles by sequencing in ox-LDL-induced THP-1 macrophages. PMA-induced THP-1 macrophages were treated with ox-LDL alone or in combination with C-1b(3-13) (6.25 μM) for 24 h. (**A**) The number of DEMs in the following: (i) ox-LDL-treated and control groups, and (ii) AMP C-1b(3-13)-treated and ox-LDL groups. |log_2_ FC| >1, *p* < 0.05. (**B**) Heatmap of differentially expressed miRNAs in the following: (i) ox-LDL-treated and control groups (left), and (ii) AMP C-1b(3-13)-treated and ox-LDL groups (right). Color scale indicated log_2_-transformed normalized expression values (red: upregulation and blue: downregulation). (**C**,**D**) Functional enrichment analysis of the predicted target genes of differentially expressed miRNAs: (**C**) KEGG enrichment pathways (level 2) and (**D**) GO terms (Level 2). (**E**) RT-qPCR validated the expression levels of the top ten differentially expressed miRNAs (*n* = 3, each point represents an independent experiment). One-way ANOVA was used to compare multiple groups for each mutation. Results are expressed as mean  ±  SEM; # *p* < 0.05, ## *p* < 0.01 vs. the control group; ** *p* < 0.01 vs. ox-LDL group.

MicroRNA sequencing was first performed to investigate the effects of AMP C-1b(3-13) on the expression level of miRNA in ox-LDL-induced THP-1 macrophages. In our previous study, C-1b(3-13) at concentrations ranging from 6.25 to 25 μM did not induce significant toxicity in ox-LDL-induced THP-1-derived foam cells, with cell viability remaining above 80%. Moreover, treatment with 6.25 μM C-1b(3-13) significantly suppressed foam cell formation [23]. Therefore, we selected 6.25 μM for subsequent miRNA sequencing and related mechanistic studies. A total of 948, 963, and 953 known miRNAs were identified in the control group, ox-LDL-induced THP-1 macrophages (ox-LDL group), and AMP C-1b(3-13)-treated ox-LDL-induced THP-1 macrophages (C-1b(3-13) group), respectively. A total of 31 miRNAs were identified with significantly differential expression in the ox-LDL group compared to the control group, including 29 upregulated and 2 downregulated ones, meeting the criteria of |log2FC| >1 with *p* < 0.05 (Figure 1A,B and Table S1). Similarly, 3 upregulated and 20 downregulated differential expression miRNAs (DEMs) were identified upon comparing AMP C-1b(3-13) with the ox-LDL group.

To functionally characterize the differentially expressed miRNAs, we predicted their putative target genes using Miranda software (v3.3a) and performed gene ontology (GO) and the Kyoto encyclopedia of genes and genomes (KEGG) pathway analysis. As illustrated in Figure 1C, the KEGG pathway analysis (Level 2) revealed that the predicted target genes were significantly enriched in the immune system (662 genes), cardiovascular disease pathways (225 genes), and lipid metabolism (196 genes). Moreover, GO analysis revealed significant enrichment in the immune system process (Figure 1D), indicating that the miRNAs modulated foam cell formation by inflammatory responses. Among the differentially expressed miRNAs (DEMs), 10 miRNAs (including miR-590-5p, miR-127-3p, miR-15b-3p, miR-126-3p, miR-142-3p, miR-181a-3p, miR-195-5p, miR-203a-3p, miR-205-5p, and miR-210-3p) exhibited significant expression changes, which were subsequently verified by RT-qPCR (Figure 1E). Notably, miR-590-5p was downregulated by 77% in ox-LDL-induced foam cells compared to the control group, while AMP C-1b(3-13) treatment reversed this effect, resulting in a 281% upregulation compared to the ox-LDL group (Figure 1E, *p* < 0.01). Therefore, we proposed that miR-590-5p functioned as a key regulator of inflammatory responses in foam cells, and AMP C-1b(3-13) attenuated foam cell formation by regulating miR-590-5p expression.

### 2.2. miR-590-5p Suppresses the Foam Cell Formation in ox-LDL-Induced PMA-THP-1 Macrophages

**Figure 2:**
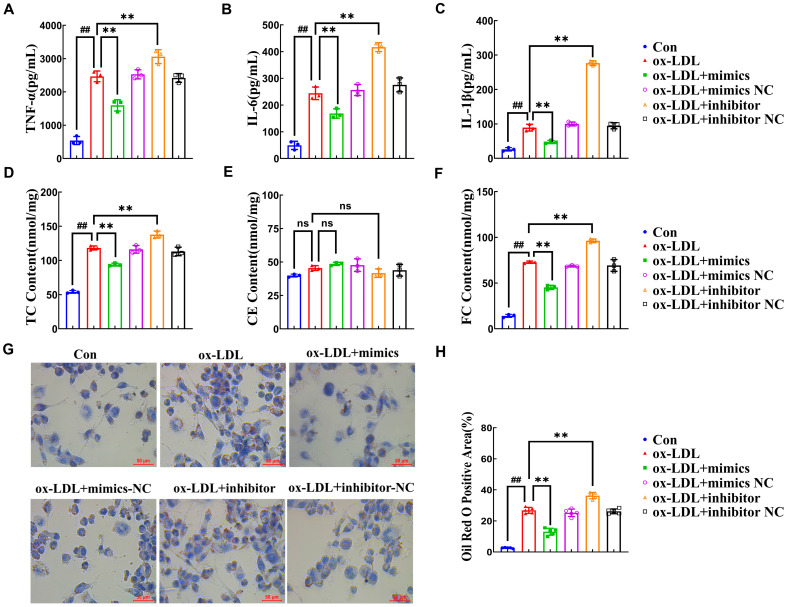
miR-590-5p attenuates inflammatory responses and lipid accumulation in ox-LDL-induced PMA-THP-1 macrophages. PMA-differentiated THP-1 macrophages were treated for 24 h as follows: control (Con) group: untreated cells; ox-LDL group: ox-LDL treatment alone; ox-LDL + miR-590-5p mimics group: ox-LDL combined with 50 nM mimics; ox-LDL + mimics negative control (NC) group: ox-LDL combined with 50 nM scrambled RNA; ox-LDL + miR-590-5p inhibitor group: ox-LDL combined with 50 nM inhibitor; ox-LDL + inhibitor negative control NC group: ox-LDL combined with 50 nM scrambled RNA. (**A**–**C**) Secretion levels of pro-inflammatory cytokines (**A**) TNF-α, (**B**) IL-6, and (**C**) IL-1β in ox-LDL-induced PMA-THP-1 macrophages measured by ELISA (*n* = 3). (**D**–**F**) Intracellular lipid content quantification: (**D**) TC, (**E**) FC, and (**F**) CE (*n* = 3). (**G**,**H**) Effect of miR-590-5p on morphological changes and lipid droplet formation in ox-LDL-induced PMA-THP-1 macrophages via ORO staining (400×) and the quantitative analysis of the corrected lipid content (red-stained area/total cell area). (*n* = 5) One-way ANOVA was used to compare multiple groups for each mutation. Results are expressed as mean ± SEM from at least three independent biological replicates. Individual data points from all independent replicates are displayed in the graphs: ns: not significant, ## *p* < 0.01 vs. control group; ns: not significant, ** *p* < 0.01 vs. ox-LDL group.

An ox-LDL-induced macrophage-derived foam cell model was first established according to our preliminary study to investigate the effect of miR-590-5p expression on the foam cell formation. First, RT-qPCR demonstrated that compared to the control group (untreated cells), ox-LDL treatment downregulated the expression of miR-590-5p by 64% (Figure S1, *p* < 0.01). Ox-LDL-induced foam cells that were transfected with miR-590-5p mimics (50 nM) increased the expression by 119%, whereas the miR-590-5p (50 nM) inhibitor reduced the expression by 70% compared to the ox-LDL group (*p* < 0.01). The negative controls (NCs: ox-LDL + mimics NC and ox-LDL + inhibitor NC), which were treated with ox-LDL and transfected with scrambled RNA, showed no significant difference in expression compared to the ox-LDL group alone. The results confirmed the specificity of the miR-590-5p mimics and the inhibitor. As presented in Figure 2A–C, ox-LDL treatment increased the release of TNF-α, IL-6, and IL-1β by 357%, 397%, and 242%, respectively, compared to the control group (*p* < 0.01). Notably, miR-590-5p overexpression suppressed TNF-α, IL-6, and IL-1β release by 35%, 33%, and 48%, respectively (Figure 2A–C, ox-LDL mimics vs. ox-LDL group, *p* < 0.01). In addition, miR-590-5p inhibition increased the pro-inflammatory cytokines to 24% (TNF-α), 71% (IL-6), and 210% (IL-1β), respectively (Figure 2A–C, ox-LDL inhibitor vs. ox-LDL group, *p* < 0.01). These results revealed that miR-590-5p overexpression significantly decreased the release of TNF-α, IL-6, and IL-1β induced by ox-LDL.

Next, the effect of miR-590-5p on lipid accumulation was evaluated in ox-LDL-induced foam cells. As shown in Figure 2D–F, total cholesterol (TC) and cholesterol ester (CE) levels significantly increased after ox-LDL treatment compared to the control group (*p* < 0.01), but free cholesterol (FC) levels showed no significant change (*p* > 0.05). However, miR-590-5p overexpression in ox-LDL-induced foam cells reduced TC and CE levels by 20% and 37% compared to the ox-LDL group, respectively, while miR-590-5p inhibition increased TC and CE by 17% and 32%, respectively (Figure 2D–F, *p* < 0.01). The overexpression and inhibition of miR-590-5p did not markedly affect the content of FC in foam cells (*p* > 0.05). Furthermore, ORO staining was used to visualize lipid droplets in ox-LDL-induced foam cells upon miR-590-5p overexpression or inhibition. Strikingly, Figure 2G–H presented that the ox-LDL group exhibited increased red-stained areas (indicating lipid droplets) compared to the control group. Notably, transfection with miR-590-5p mimics reduced this area by 51%, while miR-590-5p inhibition increased it by 35% relative to the ox-LDL group, confirming that miR-590-5p attenuates lipid deposition and its inhibition exacerbates accumulation (Figure 2G–H, *p* < 0.01). These results indicated that miR-590-5p overexpression suppressed foam cell formation.

### 2.3. miR-590-5p Alleviates Inflammatory Responses by Targeting KLF12

**Figure 3:**
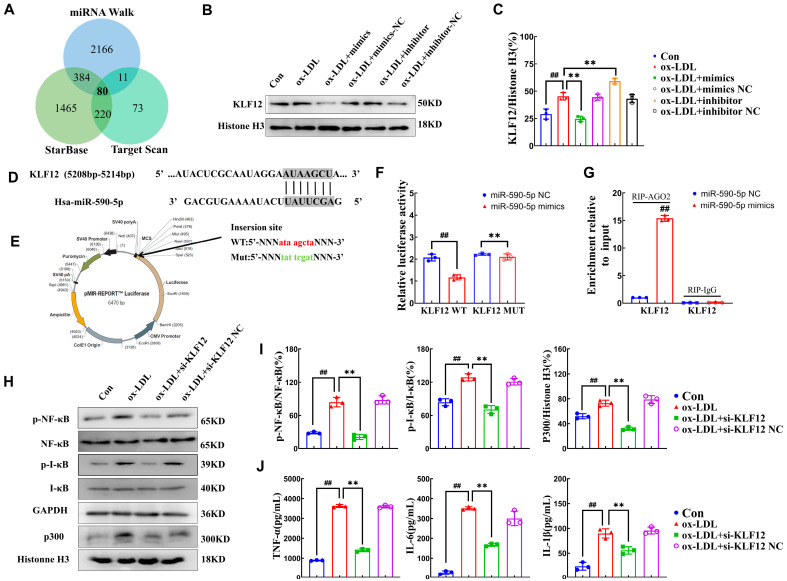
miR-590-5p alleviates inflammatory responses by targeting KLF12. (**A**) Venn diagram of predicted miR-590-5p target genes from TargetScan (v7.2), StarBase (v3.0), and miRNAWalk (v3.0). (**B**,**C**) Western blot and quantitative analysis of KLF12 protein expression in ox-LDL-induced THP-1 macrophages that were transfected with miR-590-5p mimics (50 nM) or treated with inhibitor (50 nM) (*n* = 3). (**D**) Prediction of miR-590-5p binding site in *KLF12* 3′ UTR via TargetScan (v7.2). (**E**) Schematic representation of luciferase reporter constructs: wild-type (WT) *KLF12* 3′ UTR with predicted miR-590-5p binding site (red) and mutant version with disrupted seed sequence (green). (**F**) Dual-luciferase reporter assay analyzed the miR-590-5p-induced repression of *KLF12* expression (wild-type vs. mutant 3′ UTR constructs). (**G**) RNA immunoprecipitation (RIP) assay validated the direct interaction between miR-590-5p and *KLF12* mRNA (IgG as negative control). (**H**) Effect of KLF12 knockdown on the protein levels of p-NF-κB, NF-κB, p-IκBα, IκBα (total cellular proteins), and p300 (nuclear proteins). (**I**) Quantitative analysis of the protein levels (*n* = 3). (**J**) Secreted pro-inflammatory cytokines (TNF-α, IL-6, IL-1β) following KLF12 inhibition measured by ELISA (*n* = 3). Control (Con) group: untreated cells; negative control (NC) group: received a scrambled RNA sequence (50 nM). One-way ANOVA was used to compare multiple groups for each mutation. Results are expressed as mean ± SEM from at least three independent biological replicates. Individual data points from all independent replicates are displayed in the graphs: ## *p* < 0.01 vs. control group; ** *p* < 0.01 vs. ox-LDL group.

Potential target genes of miR-590-5p were predicted by integrated analysis of three bioinformatics platforms: TargetScan (v7.2), StarBase (v3.0), and miRNAWalk (v3.0). Venn diagram analysis demonstrated 80 consensus target genes were identified (Figure 3A and Table S2). Subsequent functional annotation identified eight atherosclerosis-relevant candidate genes: transcription factors (*KLF12*, *KLF6*, *KLF9*, and *MEF2C*), epigenetic regulators (*HDAC9*), and signaling molecules (*YAP1*, *SOCS6*, and *CDC25A*). To investigate the regulatory effects of miR-590-5p on the potential target genes, RT-qPCR was performed to analyze the expression changes of eight candidate targets upon miR-590-5p overexpression or inhibition. Among these, *KLF12* exhibited the most significant alterations at the mRNA level: overexpression of miR-590-5p downregulated *KLF12* expression by 49%, while miR-590-5p inhibition upregulated *KLF12* by 97%, compared to the ox-LDL group (Figure S2, *p* < 0.01). Furthermore, Western blot analysis demonstrated that overexpression of miR-590-5p significantly reduced KLF12 protein levels by 46%, while inhibition of miR-590-5p produced the opposite effect, increasing KLF12 expression by 31% compared to the ox-LDL group (Figure 3B,C, *p* < 0.01). These results suggested that KLF12 might be a potential downstream target gene of miR-590-5p.

The *KLF12* 3′-UTR contained a conserved 7 nt sequence (AUAAGCU; positions 5208-5214) that perfectly complemented the miR-590-5p seed region (UAUUCGA), as predicted by TargetScan (v7.2) (Figure 3D). This putative interaction was validated through dual-luciferase reporter and RIP-qPCR analysis. Schematic representation of luciferase reporter constructs was presented in Figure 3E. The results showed that miR-590-5p overexpression significantly suppressed the luciferase activity of the wild-type *KLF12* 3′-UTR reporter construct by 44% (Figure 3F, ox-LDL mimics vs. ox-LDL group, *p* < 0.01), whereas mutation of the predicted binding sites abolished this inhibitory effect (*p* > 0.05). Furthermore, Ago2-based RIP assay revealed a 1465% enrichment of *KLF12* mRNA in the miR-590-5p mimics group compared to the negative control (IgG) group (Figure 3G, *p* < 0.01), conclusively identifying the 3′-UTR of *KLF12* as the functional binding site for miR-590-5p.

The canonical NF-κB signaling pathway plays a pivotal role in inflammatory responses by regulating the expression of pro-inflammatory cytokines [24]. To investigate the regulatory function of KLF12 in the NF-κB signaling pathway, we performed siRNA-mediated knockdown of KLF12 in foam cells, followed by Western blot analysis of the key NF-κB pathway components, including phosphorylated NF-κB (p-NF-κB) and IκBα (p-IκBα). As presented in Figure S3, three siRNAs targeting KLF12 were designed, and the third siRNA (si-KLF12-3) demonstrated the most potent silencing efficacy, reducing KLF12 protein levels by 73%, compared to the control group (*p* < 0.01). No significant differences were observed between the control group and the scrambled RNA negative control groups (*p* > 0.05), confirming the specificity of the siRNA.

Notably, KLF12 knockdown significantly attenuated the p-NF-κB and p-IκBα by 76% and 45%, whereas total NF-κB and IκBα protein expression remained unchanged (Figure 3H,I, ox-LDL+si KLF12 vs. ox-LDL group). Furthermore, compared to the ox-LDL group, KLF12 silencing markedly decreased the release of TNF-α, IL-6, and IL-1β by 63%, 53%, and 37%, respectively (Figure 3J, *p* < 0.05), indicating the role of KLF12 in suppressing NF-κB-mediated inflammatory responses. It has been reported that KLF12 recruits p300 to stabilize p-NF-κB binding at κB consensus sites in target gene promoters (e.g., IL-6, TNF-α, IL-1β), inducing chromatin remodeling and ultimately activating the NF-κB signaling pathway [25,26]. As shown in Figure 3H, compared to the ox-LDL group, KLF12 knockdown decreased the expression of p300 in the nucleus by 57% in foam cells (*p* < 0.05), indicating that KLF12 suppressed the NF-κB signaling pathway through p300.

### 2.4. AMP C-1b(3-13) Alleviates Inflammatory Responses in ox-LDL-Induced Foam Cells by Upregulating the Expression of miR-590-5p

**Figure 4:**
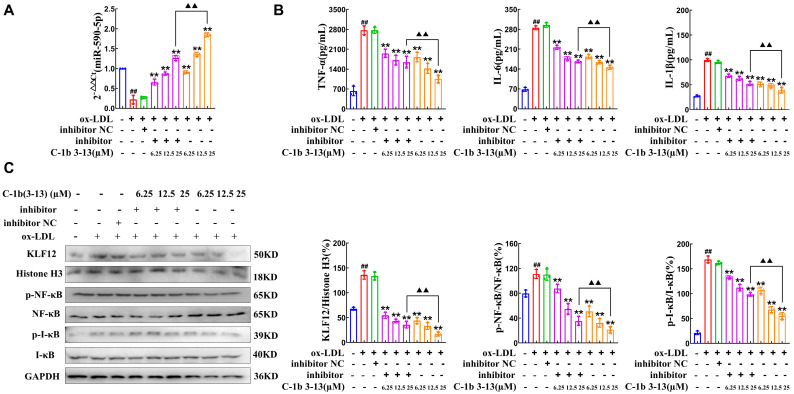
AMP C-1b(3-13) alleviates inflammatory responses in ox-LDL-induced foam cells by upregulating miR-590-5p. (**A**) RT-qPCR analysis of miR-590-5p expression in ox-LDL-induced foam cells that were treated with AMP C-1b(3-13) at concentrations ranging from 6.25 μM to 25 μM (*n* = 3). (**B**) Secretion levels of TNF-α, IL-6, and IL-1β levels in culture supernatants after the treatment with the following: (i) AMP C-1b(3-13) alone (6.25, 12.5 or 25 µM), (ii) AMP C-1b(3-13) (6.25, 12.5 or 25 µM) combined with miR-590-5p inhibitor (50 nM). (**C**) Western blot and quantitative analysis of KLF12, p300 (nuclear proteins), and p-NF-κB, NF-κB, p-IκBα, and IκBα (total cellular proteins) under the same treatments (*n* = 3). All negative controls (NCs) received a scrambled RNA sequence (50 nM). One-way ANOVA was used to compare multiple groups for each mutation. Results are expressed as mean ± SEM from at least three independent biological replicates. Individual data points from all independent replicates are displayed in the graphs: ## *p* < 0.01 vs. the control; ** *p* < 0.01 vs. the ox-LDL group; and ▲▲ *p* < 0.01 vs. the C-1b(3-13) 25 µM group.

As we had previously demonstrated that miR-590-5p inhibition alone exacerbated inflammatory responses, upregulated the KLF12 expression, and activated the NF-κB pathway (Figure 2 and Figure 3), we next determined whether the effects of C-1b(3-13) were mediated by miR-590-5p. As presented in Figure 4A,B, AMP C-1b(3-13) dose-dependently upregulated miR-590-5p expression by 308% (6.25 μM), 510% (12.5 μM), and 732% (25 μM) compared to the ox-LDL group (Figure 4A, *p* < 0.05), and a high dose (25 μM) of AMP C-1b(3-13) attenuated the release of TNF-α, IL-6, and IL-1β by 61%, 48% and 60%, respectively (Figure 4B, *p* < 0.01). Notably, the miR-590-5p inhibitor significantly reversed the inhibitory effects of AMP C-1b(3-13) on inflammatory cytokine expression (Figure 4B), revealing that the anti-inflammatory effect of C-1b(3-13) is through miR-590-5p upregulation.

To elucidate the underlying mechanism, we detected the effect of AMP C-1b(3-13) on KLF12 expression, which is a validated miR-590-5p target involved in inflammatory responses. As presented in Figure 4C, ox-LDL markedly increased KLF12 protein expression by 98%, relative to the control group (*p* < 0.05). However, the effect was dose-dependently counteracted by AMP C-1b(3-13) treatment, with the decrease of 67–87% (Figure 4C, *p* < 0.01 vs. ox-LDL group). Importantly, miR-590-5p inhibition significantly reversed the suppressive effects of AMP C-1b(3-13) on *KLF12* mRNA and protein expression (Figure 4C and Figure S4, *p* < 0.01), indicating that C-1b(3-13) suppressed KLF12 expression through miR-590-5p upregulation. Furthermore, AMP C-1b(3-13) dose-dependently reduced p-NF-κB and p-IκB levels, with the decrease of 81% and 68% at 25 μM (Figure 4C, *p* < 0.01). Notably, miR-590-5p inhibition significantly attenuated these suppressive effects. The results suggested that AMP C-1b(3-13) exerted anti-inflammatory effects by suppressing the KLF12 expression via miR-590-5p in foam cells.

### 2.5. AMP C-1b(3-13) Inhibits AS in ApoE^−/−^ Mice via the miR-590-5p/KLF12/p300 Axis

**Figure 5:**
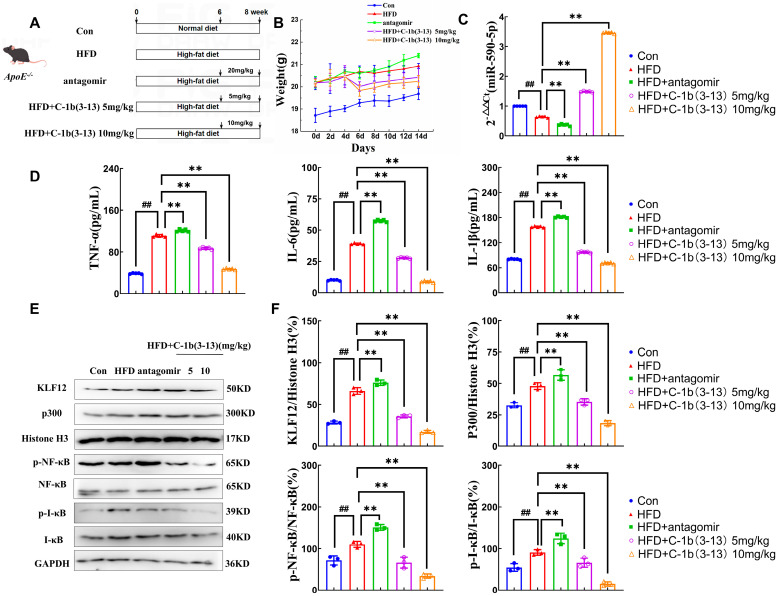
AMP C-1b(3-13) inhibits AS in *ApoE*^−/−^ mice Via the miR-590-5p/KLF12/p300 axis. (**A**) Experimental design and workflow. Con (Control) group: *ApoE*^−/−^ mice fed on a normal diet; HFD group: *ApoE*^−/−^ mice fed a high-fat diet; HFD and antagomir: HFD-induced *ApoE*^−/−^ mice that were treated with miR-590-5p antagomir (20 mg/kg) to suppress miR-590-5p expression; HFD + C-1b(3-13), 5 or 10 mg/kg: HFD-induced *ApoE*^−/−^ mice injected with AMP C-1b(3-13) at 5 or 10 mg/kg. (**B**) Effect of the AMP C-1b(3-13) on body weight in *ApoE*^−/−^ mice (*n* = 15). (**C**) RT-qPCR analysis of miR-590-5p expression of aortic arch in *ApoE*^−/−^ mice (*n* = 5). (**D**) Secretion levels of the pro-inflammatory cytokines TNF-α, IL-6, and IL-1β in plasma from *ApoE*^−/−^ mice (*n* = 5). (**E**,**F**) Western blot and quantitative analysis of KLF12, p300 (nuclear proteins), and p-NF-κB, NF-κB, p-IκBα, and IκBα (total cellular proteins) of the aortic arch in *ApoE*^−/−^ mice (*n* = 3). One-way ANOVA was used to compare multiple groups for each mutation. Results are expressed as mean  ±  SEM from at least three independent biological replicates. Individual data points from all independent replicates are displayed in the graphs: ## *p* < 0.01 vs. Con; ** *p* < 0.01 vs. the HFD group.

To evaluate the effect of AMP C-1b(3-13) in vivo, a high-fat diet(HFD)-induced AS model in *ApoE*^−/−^ mice was initially established, followed by treatment of AMP 3-13 or antagomir (to inhibit miR-590-5p expression) (Figure 5A). The doses of AMP C-1b(3-13) (5 and 10 mg/kg) were selected based on our previous results that 10 mg/kg significantly inhibited atherosclerotic plaque formation without inducing hepatic injury or other toxic side effects [23]. The 5 mg/kg dose was additionally included to explore the dose–response relationship. Following 6 weeks of HFD feeding, the treatment of AMP C-1b(3-13) (5 or 10 mg/kg) for 2 weeks significantly attenuated HFD-induced weight gain, whereas antagomir (20 mg/kg) increased body weight, compared to the HFD group (Figure 5B, *p* < 0.01). In addition, the effect of AMP C-1b(3-13) on miR-590-5p expression was detected by RT-qPCR. The results demonstrated that HFD reduced miR-590-5p expression by 36% in aortic arches compared with the control group (Figure 5C, *p* < 0.01). In contrast, AMP C-1b(3-13) treatment dose-dependently restored miR-590-5p expression, with the increased expression of 133% (5 mg/kg) and 443% (10 mg/kg), relative to the HFD group (Figure 5C, *p* < 0.01). Furthermore, the high-dose AMP C-1b(3-13) (10 mg/kg) significantly suppressed inflammation, reducing plasma levels of IL-6 (78%), IL-1β (55%), and TNF-α (58%), compared to HFD group (Figure 5D, *p* < 0.01), indicating that miR-590-5p overexpression induced by AMP C-1b(3-13) suppressed inflammatory responses in vivo.

Moreover, the regulatory effect of AMP C-1b(3-13) on the expression of KLF12 (the target of miR-590-5p) and the key proteins of the KLF12/p300 axis in aortic arches was detected by Western blot. The results demonstrated that HFD upregulated KLF12 protein by 134% (compared to the control group), while the miR-590-5p inhibition through antagomir treatment further elevated KLF12 levels by 10% compared to the HFD group (Figure 5E,F compares the levels to the HFD group, *p* < 0.01). In contrast, AMP C-1b(3-13) administration dose-dependently attenuated KLF12 upregulation, with the 10 mg/kg dose achieving a 74% reduction relative to HFD control (*p* < 0.01). Notably, the high-dose AMP C-1b(3-13) (10 mg/kg) also significantly inhibited p300, p-NF-κB, and p-IκB levels by 61%, 69%, and 83%, respectively (Figure 5E,F: AMP C-1b(3-13) vs. HFD group, *p* < 0.01). These findings demonstrated that AMP C-1b(3-13) exerted the anti-inflammatory effects in atherosclerotic *ApoE*^−/−^ mice Via miR-590-5p/KLF12/p300 signaling axis.

**Figure 6:**
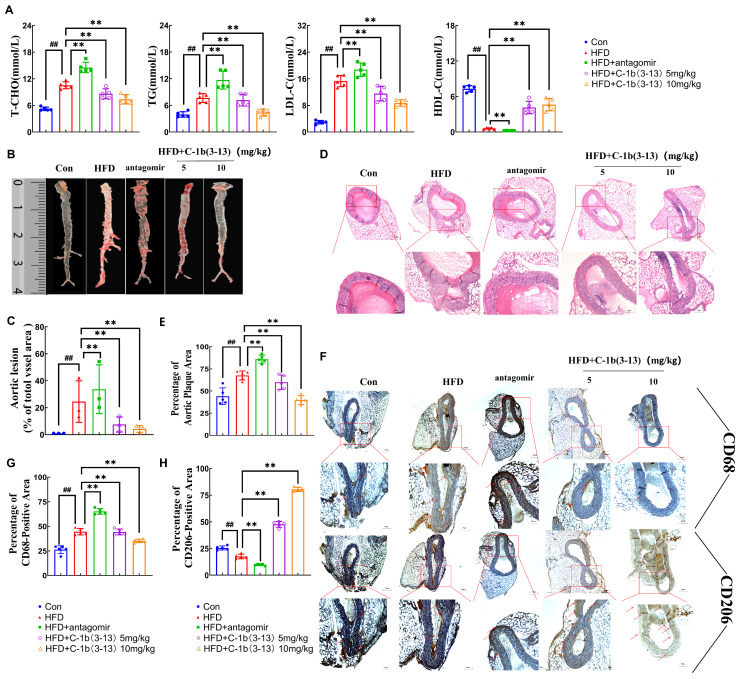
Therapeutic effects of AMP C-1b(3-13) Via miR-590-5p in atherosclerotic *ApoE*^−/−^ mice. (**A**) Plasma levels of TC, LDL-C, TG, and HDL-C in *ApoE*^−/−^ mice (*n* = 5). (**B**,**C**) ORO staining of the aortic root and quantitative analysis of the corrected plaque areas in *ApoE*^−/−^ mice (*n* = 3). (**D**) H&E staining of the aortic root vessels in *ApoE*^−/−^ mice (*n* = 5). (**E**) Quantitative analysis of H&E staining in the aortic arch (red aortic plaque area / total vessel wall area). (*n* = 5) (**F**) Immunohistochemical staining analysis of pro-inflammatory CD68+ M1 macrophages and anti-inflammatory CD206+ M2 macrophages of the aortic root in *ApoE*^−/−^ mice (*n* = 5). (**G**,**H**) Quantitative analysis of CD68+ and CD206+ areas in the aortic arch (CD68+ -positive area and CD206+ -positive area / total vessel wall area). (*n* = 5) One-way ANOVA was used to compare multiple groups for each mutation. Results are expressed as mean  ±  SEM from at least three independent biological replicates. Individual data points from all independent replicates are displayed in the graphs: ## *p* < 0.01 vs. the control group; ** *p* < 0.01 vs. the ox-LDL group. Red arrow: representative positive result.

The effect of AMP C-1b(3-13) on plasma lipids, including total cholesterol (T-CHO), triglyceride (TG), high-density lipoprotein cholesterol (HDL-C), and low-density lipoprotein cholesterol (LDL-C), was detected in *ApoE*^−/−^ mice. Relative to the control group, HFD-fed *ApoE*^−/−^ mice markedly increased T-CHO, TG, and LDL-C by 102%, 94%, and 419%, respectively, which were also exacerbated by miR-590-5p antagomir treatment (Figure 6A, *p* < 0.01). In contrast, high-dose AMP C-1b(3-13) significantly reduced the content of T-CHO, TG, and LDL-C by 31%, 45%, and 44%, while remarkably increasing HDL-C by 663% compared to the HFD group (Figure 6A, *p* < 0.01).

Quantitative analysis of ORO staining revealed a 2354% increase in lipid-rich plaque area in the aortic root of HFD-fed mice compared to the control group (*p* < 0.01), whereas the treatment of 10 mg/kg AMP C-1b(3-13) reduced plaque area by 85%, relative to the HFD group (Figure 6B,C, *p* < 0.01). H&E-stained aortic root sections revealed that the plaque area in the aortic root of HFD-fed mice increased by 51% compared to the control group. Relative to the HFD group, miR-590-5p antagomir further increased the plaque area by 29%, whereas administration of AMP C-1b(3-13) effectively attenuated this increase (Figure 6D,E, *p* < 0.01). Immunohistochemical analysis revealed that HFD significantly increased infiltration of pro-inflammatory CD68+ M1 macrophages by 73%, while decreasing anti-inflammatory CD206+ M2 macrophages by 31% (Figure 6F–H, *p* < 0.01 vs. control group). Moreover, antagomir treatment exhibited similar effects to HFD. In contrast, AMP C-1b(3-13) treatment reversed this imbalance, significantly reducing M1 macrophage infiltration while increasing M2 macrophage, compared to the HFD group (Figure 6F–H, *p* < 0.01). These results demonstrated that AMP C-1b(3-13) inhibited the atherosclerotic plaque formation through regulating macrophage polarization and inflammatory responses in *ApoE*^−/−^ mice.

## 3. Discussion

Atherosclerosis is a chronic inflammatory disease of the arterial wall characterized by lipid accumulation, inflammatory cascades, and extracellular matrix within the intimal layer [27]. This pathological process leads to the formation of plaques that can restrict blood flow or rupture to cause acute thrombosis [28]. Recent studies highlight the diagnostic potential of circulating miRNAs due to their remarkable stability in bodily fluids, tissue-specific expression profiles, and dynamic correlation with disease progression and therapeutic response [29]. Notably, specific miRNA families demonstrate distinct regulatory roles in atherogenic processes: The miR-33a/b cluster potently suppresses reverse cholesterol transport via post-transcriptional repression of ABCA1 and ABCG1 transporters, whereas miR-122 exacerbates intracellular lipid accumulation through direct targeting of fatty acid synthase [30,31]. Particularly, miR-155 and miR-146a emerge as master regulators of macrophage phenotypic switching, regulating inflammatory responses through their respective downstream targets-Bcl6 (for miR-155) and the TRAF6/IRAK1 signaling axis (for miR-146a) [32,33]. MiR-590-5p, a member of the microRNA-590 family located on chromosome 7q11.23, is implicated in tumor proliferation, attenuation of intestinal inflammation, and repair of hemorrhagic brain injury [34,35,36]. Notably, in endothelial cells, it inhibits angiogenesis by targeting LOX-1 and suppressing redox-sensitive signaling, thereby delaying atherosclerotic progression [37]. These findings suggest a key regulatory role for miR-590-5p in atherosclerosis. However, its function in inflammatory signaling during foam cell formation is still not fully understood. In addition, our previous work demonstrated that AMP C-1b(3-13), which is a truncated analog of Chensinin-1b, reduced macrophage-derived foam cell formation and alleviated atherosclerosis [23,38]. This study investigated the effects of miR-590-5p on foam cell formation and further elucidated a novel mechanism through which AMP C-1b(3-13) attenuates atherosclerotic progression by modulating miR-590-5p-dependent pathways.

To elucidate the role of AMP C-1b(3-13) in AS, miRNA sequencing was first performed in ox-LDL-induced macrophages, and miR-590-5p was identified as a miRNA markedly downregulated during foam cell formation, an effect that was reversed by AMP C-1b(3-13) treatment. Functional assays confirmed that miR-590-5p upregulation suppressed both inflammatory cytokine secretion and lipid accumulation, whereas its inhibition exacerbated the pathological features. We further identified KLF12 as a direct target of miR-590-5p, and observed its elevated expression in foam cells and atherosclerotic aortae of *ApoE^−/−^* mice, which was reversed by miR-590-5p overexpression. Mechanistically, KLF12 recruited p300 to stabilize p-NF-κB and promote pro-inflammatory gene expression. We demonstrated that miR-590-5p-mediated KLF12 silencing disrupted p300 recruitment, thereby inhibiting NF-κB signaling. Importantly, AMP C-1b(3-13) dose-dependently elevated miR-590-5p levels and suppressed inflammation in an miR-590-5p-dependent manner both in vitro and in vivo. Collectively, this study elucidates the mechanism through which the AMP C-1b(3-13) modulates the miR-590-5p/KLF12/p300 signaling axis in AS, thereby revealing the role of antimicrobial peptides in cardiovascular diseases. Furthermore, KLF12 is identified as a key downstream effector of miR-590-5p, expanding the known biological functions of the KLF protein family in cardiovascular pathophysiology and providing a novel theoretical foundation for targeted AS therapy.

However, it is important to note that the underlying mechanisms have not been fully elucidated. Firstly, why does a frog skin-derived peptide exert such effects? The anti-atherosclerotic activity of AMP C-1b(3-13) derives from its evolutionary origin as a host defense peptide (HDP). The HDP derived from frog skin originally regulates immune responses to rapidly combat microbial invasions and tissue damage [39]. Many of the skin-derived peptides inherently exhibit anti-inflammatory properties [40]. In atherosclerosis, which is a chronic inflammatory disease, the peptides may exert their immunomodulatory functions by suppressing macrophage-mediated inflammatory responses, thereby demonstrating anti-atherosclerotic effects. Secondly, although our study delineates the downstream miR-590-5p/KLF12/p300 pathway, the initial binding partner of AMP C-1b(3-13) remains to be definitively identified. As we previously reported, AMP C-1b(3-13) is distributed in both the cytoplasm and nucleus of foam cells [23], suggesting that the peptides may function through intracellular target proteins. We hypothesized that the primary target is likely a protein involved in the transcriptional regulation or maturation of miR-590-5p. Potential candidates include transcription factors that govern the pri-miR-590 promoter, or core components of the microprocessor (e.g., Drosha/DGCR8) and Dicer complexes. The definitive identification of this initial interactor is a central objective of our future research, which will employ affinity pull-down coupled with mass spectrometry and functional genetic screens to precisely identify the binding entity.

Moreover, several limitations of this study should be considered. First, the long-term pharmacokinetics, potential immunogenicity, and sustained efficacy of AMP C-1b(3-13) require comprehensive evaluation and optimization. AMP C-1b(3-13) is a short peptide of 11 amino acids that generally exhibits low immunogenicity, primarily due to its small molecular size, therefore minimizing the risk of inducing an adaptive immune response and antibody formation [41,42]. The low immunogenicity also contributes to a reduced probability of resistance development after long-term use [43]. A decade of research in our laboratory on the AMP chensinin-1b and its analogs has demonstrated its low immunogenicity and inability to induce drug resistance, after two to four weeks of treatment [22,23,38,44]. Nonetheless, the potential risks remain a possibility that cannot be excluded. Future studies directly detected the long-term repeated dosing in *ApoE*^−/−^ mice to monitor for efficacy loss, serum analysis for anti-drug antibodies, and investigation of potential adaptations in the miR-590-5p/KLF12 axis following chronic treatment. Secondly, a limitation is our incomplete understanding of the precise in vivo target cells for both miR-590-5p and AMP C-1b(3-13). In this study, we focused on macrophage-derived foam cells at the aortic plaque and demonstrated that AMP C-1b(3-13) suppressed inflammation by upregulating miR-590-5p and inhibiting the KLF12/NF-κB pathway in the cells. Therefore, we proposed that foam cells at the aortic plaque are one of the cellular targets of AMP C-1b(3-13) and miR-590-5p in vivo. However, the pathogenesis of atherosclerosis involves multiple cell types in addition to macrophage-derived foam cells, such as endothelial cells and vascular smooth muscle cells. Whether the observed therapeutic effects are primarily mediated through macrophage-derived foam cells at the aortic plaque or result from coordinated actions across multiple cell types warrants further investigation. These efforts will help develop novel strategies for the prevention and treatment of cardiovascular diseases.

## 4. Materials and Methods

### 4.1. Peptide Synthesis

The frog skin AMP C-1b(3-13) was synthesized by GL Biochemistry (Shanghai, China) with >95% purity. Its molecular mass was confirmed by MALDI-TOF MS (Shimadzu, Kyoto, Japan).

### 4.2. Cell Culture

THP-1 human monocytic leukemia cells (Jiangsu KaiJi Bio, Nanjing, China) were maintained in RPMI-1640 with 10% FBS, 100 µg/mL penicillin, and 100 µg/mL erythromycin (37 °C, 5% CO_2_). Macrophage differentiation was induced by phorbol-12-myristate-13-acetate (PMA; Sigma-Aldrich, Burlington, MA, USA) and mesityl-OAc (Sigma-Aldrich). Cells were seeded at 1 × 10^6^ cells/well in 6-well plates for 24 h, then differentiated with 100 ng/mL PMA for 48 h. Differentiated macrophages were exposed to 100 µg/mL ox-LDL (24 h) prior to CCK-8 assays and miR-590-5p expression analysis via RT-qPCR.

### 4.3. MicroRNA Sequencing (miRNA-Seq) and Data Analysis

THP-1 cells were primed with 100 ng/mL PMA (48 h) in 10 cm dishes (1 × 10^5^ cells/mL, 6 mL/dish) and allocated to the following: (1) control: standard culture; (2) ox-LDL: 100 μg/mL ox-LDL; and (3) C-1b(3-13): co-treated with 100 µg/mL ox-LDL and 6.25 µM C-1b(3-13). After 24 h treatment, cells underwent lysis in 1.5 mL ice-cold TRIzol, snap-freezing in liquid N_2_, and dry ice shipment to Oebiotech Co., Ltd. (Shanghai, China) post-QC. Bioinformatics analysis encompassed differential miRNA expression profiling, target gene prediction, GO enrichment, and KEGG pathway annotation.

### 4.4. Cell Transfection

PMA-induced THP-1 cells were transfected using Lipofectamine^TM^ 3000 (Thermo Fisher Scientific, Waltham, MA, USA) using the following nucleic acids: miR-590-5p mimics, the 2′-O-methyl-modified miR-590-5p inhibitor, and si-KLF12 (Sangon Biotech, Shanghai, China). Scrambled sequences were used as negative controls for each respective molecule. Briefly, nucleic acids and transfection reagent were separately diluted in Opti-MEM^TM^ (Meilun Bio, Dalian, China), combined, and incubated (20 min, room temperature). After medium replacement with Opti-MEM^TM^ reduced-serum medium, transfection complexes were added, and cells were incubated (6 h, 37 °C, 5% CO_2_). The medium was then replaced with complete medium, and cells were cultured for 24 h before harvest. The sequences of all oligonucleotides used are provided in Table S3 of the Supplementary Materials.

### 4.5. Cholesterol Detection

PMA-induced THP-1 cells were treated with ox-LDL in the absence and presence of C-1b(3-13) or miR-590-5p mimics (50 nM) or miR-590-5p inhibitor (50 nM) for 24 h. Cells were lysed, and free cholesterol (FC) and total cholesterol (TC) levels were assayed using commercial kits (Solarbio, Shanghai, China) per the manufacturer’s guidelines. Protein content was determined by BCA assay (Meilun Bio), with FC and TC normalized relative to protein. Cholesterol ester (CE) content was derived as follows: CE = TC − FC.

### 4.6. Oil Red O Staining

Oil Red O staining (Solarbio) was employed to visualize lipid droplets in ox-LDL-stimulated macrophages. Briefly, PMA-induced THP-1 cells were seeded in 24-well plates, incubated for 24 h, and then treated with ox-LDL in the absence and presence of miR-590-5p mimics (50 nM) or miR-590-5p inhibitor (50 nM) for an additional 24 h. Post-treatment, cells were PBS-washed, fixed in ORO fixative (20 min), permeabilized with 60% isopropanol (5 min), then stained with ORO working solution and hematoxylin counterstain. Micrographs were acquired using a Leica light microscope (Wetzlar, Germany).

### 4.7. ELISA Detection

PMA-induced THP-1 cells were treated with ox-LDL in the absence and presence of C-1b(3-13) or miR-590-5p mimics or miR-590-5p inhibitor for 24 h, and were analyzed for TNF-α, IL-6, and IL-1β using commercial ELISA kits (Neobioscience, Shenzhen, China) per the manufacturer’s protocols. Absorbance was measured on a Multiskan FC microplate reader (Thermo Fisher Scientific).

### 4.8. Quantitative Real-Time PCR

PMA-induced THP-1 cells were treated with ox-LDL in the absence and presence of C-1b(3-13) or miR-590-5p mimics or miR-590-5p inhibitor for 24 h. Total RNA was extracted with TRIzol reagent (Invitrogen, Shanghai, China). cDNA synthesis employed SuperScript™ III (Invitrogen), followed by qPCR amplification on an ABI 7500 Fast system (Applied Biosystems, San Francisco, CA, USA) with SYBR Premix Ex Taq II (TaKaRa, Dalian, China). Gene expression quantification normalized miR-590-5p to U6, and KLF12 to GAPDH, using the 2^−ΔΔCt^ method.

### 4.9. Western Blot

PMA-induced THP-1 cells were treated with ox-LDL in the absence and presence of C-1b(3-13) or miR-590-5p mimics or miR-590-5p inhibitor for 24 h, cells were washed with ice-cold PBS and pelleted by centrifugation (12,000 rpm, 10 min). Total protein was extracted using RIPA lysis buffer (Thermo Fisher Scientific) containing 1× protease/phosphatase inhibitor cocktail (MedChemExpress, Shanghai, China). Protein concentration was quantified via the Qubit™ Protein BR Assay Kit (Thermo Scientific). Proteins were resolved by SDS-PAGE (concentrating gel: 80 V and separating gel: 120 V) and transferred to PVDF membranes (Millipore, Beijing, China) via semi-dry electrophoresis. After 2 h blocking in TBST (20 mM Tris pH 7.5, 150 mM NaCl, 0.1% Tween 20) with 5% skim milk, membranes were probed overnight at 4 °C with primary antibodies against the following: I-κBα (A19714, Abclonal, Wuhan, China, 1:1000 dilution), p-I-κBα (AP0707, Abclonal, 1:1000 dilution, phospho-Ser32), NF-κB p65 (A6667, Abclonal, 1:1000 dilution), p-NF-κB p65 (AP0125, Abclonal, 1:1000 dilution, phospho-Ser337), KLF12 (sc-134373, Santa Cruz Biotechnology, Dallas, TX, USA, 1:1000 dilution), p300 (bs-6954R, Bioss, 1:1000 dilution), GAPDH (81640-5-RR, Proteintech, Chicago, IL, USA, 1:20,000 dilution), and Histone H3 (17168-1-AP, Proteintech, 1:5000 dilution). After TBST washes, membranes were incubated with HRP-conjugated anti-rabbit IgG (1:2000, 40 min, RT), followed by ECL development (Tanon, Shanghai, China). Signal detection used an Azure c500 system (Azure Biosystems, Dublin, CA, USA) with densitometric analysis in ImageJ (v1.25a), normalized to GAPDH or Histone H3.

### 4.10. miRNA Target Prediction and Bioinformatics Analyses

The target gene of miR-590-5p was predicted by using the miRNA target prediction websites TargetScan, StarBase, and miRNAWalk. Based on the conserved complementary matching between the miRNA seed region (positions 2-8 nt) and the 3′UTR of target genes, potential binding sites of miR-590-5p were identified. A binding free energy threshold (ΔG ≤ −20 kcal/mol) was applied, and only target genes consistently predicted by all three algorithms were retained. Subsequent functional analysis was performed on the screened target genes of miR-590-5p.4.10. Peptide synthesis was also assessed.

### 4.11. Dual-Luciferase Reporter Assay

Dual-luciferase reporter assays were performed to investigate whether KLF12 is a direct downstream target gene of miR-590-5p. According to the TargetScan website (https://www.targetscan.org/vert_72/, accessed on 1 May 2023), the wild-type 3′-UTR of the KLF12 gene contains the complementary sequence of miR-590-5p. So, the wild-type and corresponding mutational 3′-UTR of the KLF12 gene were synthesized and cloned into the dual luciferase reporter vector pMIR-REPORTTM-Luciferase (GenePharma, Shanghai, China). Following the manufacturer’s instructions, the reporter vector was co-transfected into HEK293T cells expressing miR-590-5p mimics (50 nM), or miR-590-5p inhibitor (50 nM), or mimic NC, or inhibitor NC. The relative luciferase activity was assessed using a Dual Luciferase Reporter Assay System (Promega, Shanghai, China) at 48 h post-transfection, and the results were normalized to the human kidney luciferase activity.

### 4.12. RNA Immunoprecipitation

Briefly, PMA-induced THP-1 cells were treated with ox-LDL in the absence and presence of miR-590-5p mimics (50 nM) or mimics NC for 24 h. After the cells were lysed, cell lysates were used for immunoprecipitation by using Magna RIP™ RNA-Binding Protein Immunoprecipitation Kit (Millipore, Billerica, MA, USA) according to the manufacturer’s instructions. Anti-Argonaute2 (anti-Ago2) (ab32381, Abcam, Cambridge, UK) and anti-IgG antibody (ab133470, Abcam) were used to co-precipitate the RNA-binding proteins of miR-590-5p. Cell lysate was co-incubated with A/G magnetic beads together with anti-Ago2 or anti-IgG antibody. After proteinase K digestion, the enrichment of UCA1 and miR-590-5p was detected by RT-qPCR assay.

### 4.13. Animal Model

*ApoE^−/−^* male mice that were 6 weeks old were used to establish an AS model by high-fat diet (HFD) feeding. After 6 weeks of HFD feeding, the mice were randomly divided into five groups: (1) control group (Con); (2) HFD; (3) miR-590-5p antisense group (Antagomir); (4) 5 mg/kg AMP C-1b(3-13) group (C-1b(3-13)-L); and (5) 10 mg/kg AMP C-1b(3-13) group (C-1b(3-13)-H). Each group contained 15 mice. Antagomir and AMP C-1b(3-13) (dissolved in 0.9% physiological salt solution) were administered via intraperitoneal injections for 2 weeks. All mice were housed in independent humidity-controlled cages (22 °C, 50–60% humidity) with standard 12 h light and 12 h dark cycles, and the activity status and behavioral changes in the mice were carefully observed and recorded.

### 4.14. ORO Staining of the Mouse Aorta

Following cervical dislocation euthanasia, mice were secured on an operating platform. The aortic arch was microsurgically isolated through blunt dissection of abdominal/thoracic cavities under stereomicroscopic guidance, with removal of thymus, esophagus, and trachea. Excised aortae were saline-rinsed, fixed in 4% paraformaldehyde (RT, 10 min), and cleared of adventitial adipose tissue under microscopy. Tissues were stained in pre-warmed 0.3% ORO (45 °C, 10 min, sealed container), differentiated in 75% ethanol until arterial walls regained milky-white appearance, saline-washed, and imaged on black absorbent paper.

### 4.15. Immunohistochemical Staining

Aortic arches from five mice were subjected to consecutive sectioning. Tissue sections underwent deparaffinization and rehydration via graded ethanols, followed by microwave-mediated antigen retrieval in citrate buffer. Endogenous peroxidase was quenched with 3% H_2_O_2_ (15 min, RT), and nonspecific sites were blocked with 5% normal goat serum. Sections were incubated overnight at 4 °C with PBS-diluted primary antibodies, then with HRP-conjugated secondary antibodies (1 h, 37 °C). DAB development preceded hematoxylin counterstaining, with final dehydration through graded alcohols, xylene clearance, and neutral resin mounting for imaging.

### 4.16. Histopathological Analysis

Following a 14-day drug treatment, 5 mice were randomly selected, and their aortic arches were dissected and subjected to sequential ethanol dehydration: 75% (1.5 h), 80% (1.5 h), 90% (1 h), 95% (30 min), followed by two changes of 100% ethanol (20 min each) and 1:1 ethanol:xylene (20 min). Tissues were cleared in xylene (20 min), paraffin-embedded, and sectioned using a Leica CM1950 microtome. Histopathological alterations were evaluated in H&E-stained sections across experimental groups, with representative micrographs documented.

### 4.17. Statistical Analysis

Data are presented as the mean ± standard error of the mean (SEM) from at least three independent biological replicates. Individual data points from all independent replicates are displayed in the graphs. Statistical significance between groups was determined using an unpaired, two-tailed Student’s *t*-test (for comparisons of two groups) or one-way ANOVA followed by an appropriate post hoc test (for comparisons of multiple groups). The values were considered significant when *p* < 0.05.

## 5. Conclusions

**Figure 7:**
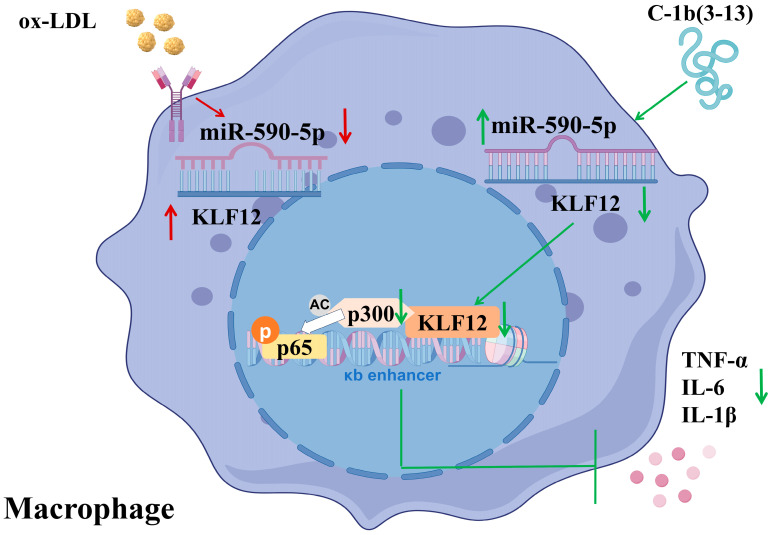
Schematic diagram of the molecular mechanism by which antimicrobial peptide C-1b(3-13) inhibits atherosclerosis via the miR-590-5p-regulated KLF12/p300 axis. AMP C-1b(3-13) upregulates miR-590-5p, which directly binds to the 3′UTR of *KLF12* mRNA, thereby suppressing KLF12 expression. KLF12 downregulation reduces nuclear accumulation of p300, consequently attenuating NF-κB signaling pathway activation. Consequently, AMP C-1b(3-13) alleviates inflammatory responses in foam cells and suppresses aortic plaque formation. Red arrows: pathology; Green arrows: AMP treatment.

In conclusion, this study identified KLF12 as a novel downstream effector of miR-590-5p in the pathogenesis of AS, thereby unveiling a previously unrecognized regulatory axis—miR-590-5p/KLF12/p300—that governed inflammation and foam cell formation. These findings significantly expanded the known functional repertoire of miR-590-5p in cardiovascular disease, highlighting its dual role as both a biomarker and a therapeutic target. In addition, we demonstrated that the AMP C-1b(3-13) exerted anti-atherosclerotic effects by upregulating miR-590-5p, consequently suppressing KLF12-mediated NF-κB signaling pathway. The ability of AMP C-1b(3-13) to attenuate plaque burden and inflammatory responses in *ApoE*^−/−^ mice positioned it as a promising candidate for AS therapeutic intervention (Figure 7).

## Data Availability

The original contributions presented in this study are included in the article/Supplementary Material. Further inquiries can be directed to the corresponding author.

## Supplementary Materials

The following supporting information can be downloaded at: https://www.mdpi.com/article/10.3390/ijms262311497/s1.

## Abbreviations

The following abbreviations are used in this manuscript:

AMP	antimicrobial peptide
CE	cholesterol ester
DEMs	differential expression miRNAs
FATP	fatty acid transport protein
FC	free cholesterol
GO	gene ontology
H&E	hematoxylin and eosin
HDL-C	high-density lipoprotein cholesterol
HFD	high-fat diet
KEGG	Kyoto encyclopedia of genes and genomes
KLF12	Krüppel-like factor 12
LDL-C	low-density lipoprotein cholesterol
LPL	lipoprotein lipase
MAPK	mitogen-activated protein kinase
miRNAs	microRNAs
miRNA-Seq	MicroRNA sequencing
NF-κB	nuclear factor kappa B
ORO staining	oil red O staining
ox-LDL	oxidized low-density lipoprotein
PMA	phorbol 12-myristate 13-acetate
RIP	RNA immunoprecipitation
TC	total cholesterol
T-CHO	total cholesterol
TG	triglyceride

## References

[1] Koelwyn G.J., Corr E.M., Erbay E., Moore K.J. (2018). Regulation of macrophage immunometabolism in atherosclerosis. Nat. Immunol..

[2] Yu X.H., Fu Y.C., Zhang D.W., Yin K., Tang C.K. (2013). Foam cells in atherosclerosis. Clin. Chim. Acta.

[3] Reustle A., Torzewski M. (2018). Role of p38 MAPK in Atherosclerosis and Aortic Valve Sclerosis. Int. J. Mol. Sci..

[4] Yu X.H., Zheng X.L., Tang C.K. (2015). Nuclear Factor-κB Activation as a Pathological Mechanism of Lipid Metabolism and Atherosclerosis. Adv. Clin. Chem..

[5] Wang D., Yang Y., Lei Y., Tzvetkov N.T., Liu X., Yeung A.W.K., Xu S., Atanasov A.G. (2019). Targeting Foam Cell Formation in Atherosclerosis: Therapeutic Potential of Natural Products. Pharmacol. Rev..

[6] Jebari-Benslaiman S., Galicia-García U., Larrea-Sebal A., Olaetxea J.R., Alloza I., Vandenbroeck K., Benito-Vicente A., Martín C. (2022). Pathophysiology of Atherosclerosis. Int. J. Mol. Sci..

[7] Herrington W., Lacey B., Sherliker P., Armitage J., Lewington S. (2016). Epidemiology of Atherosclerosis and the Potential to Reduce the Global Burden of Atherothrombotic Disease. Circ. Res..

[8] Ono K., Horie T., Nishino T., Baba O., Kuwabara Y., Kimura T. (2015). MicroRNAs and High-Density Lipoprotein Cholesterol Metabolism. Int. Heart J..

[9] Moreno-Moya J.M., Vilella F., Simón C. (2014). MicroRNA: Key gene expression regulators. Fertil. Steril..

[10] Zhang M., Wu J.F., Chen W.J., Tang S.L., Mo Z.C., Tang Y.Y., Li Y., Wang J.L., Liu X.Y., Peng J. (2014). MicroRNA-27a/b regulates cellular cholesterol efflux, influx and esterification/hydrolysis in THP-1 macrophages. Atherosclerosis.

[11] Rotllan N., Zhang X., Canfrán-Duque A., Goedeke L., Griñán R., Ramírez C.M., Suárez Y., Fernández-Hernando C. (2022). Antagonism of miR-148a attenuates atherosclerosis progression in *APOB^TG^Apobec*^−/−^*Ldlr*^+/−^ mice: A brief report. Biomed. Pharmacother..

[12] Zhu J., Liu B., Wang Z., Wang D., Ni H., Zhang L., Wang Y. (2019). Exosomes from nicotine-stimulated macrophages accelerate atherosclerosis through miR-21-3p/PTEN-mediated VSMC migration and proliferation. Theranostics.

[13] Holland A., Enrick M., Diaz A., Yin L. (2023). Is miR-21 A Therapeutic Target in Cardiovascular Disease?. Int. J. Drug Discov. Pharm..

[14] He W., Zhao L., Wang P., Ren M., Han Y. (2025). MiR-125b-5p ameliorates ox-LDL-induced vascular endothelial cell dysfunction by negatively regulating TNFSF4/TLR4/NF-κB signaling. BMC Biotechnol..

[15] Lv B., He S., Li P., Jiang S., Li D., Lin J., Feinberg M.W. (2024). MicroRNA-181 in cardiovascular disease: Emerging biomarkers and therapeutic targets. FASEB J..

[16] Erdem Büyükkiraz M., Kesmen Z. (2022). Antimicrobial peptides (AMPs): A promising class of antimicrobial compounds. J. Appl. Microbiol..

[17] Bucataru C., Ciobanasu C. (2024). Antimicrobial peptides: Opportunities and challenges in overcoming resistance. Microbiol. Res..

[18] Luo Y., Song Y. (2021). Mechanism of Antimicrobial Peptides: Antimicrobial, Anti-Inflammatory and Antibiofilm Activities. Int. J. Mol. Sci..

[19] Lee J., Lee D.G. (2015). Antimicrobial Peptides (AMPs) with Dual Mechanisms: Membrane Disruption and Apoptosis. J. Microbiol. Biotechnol..

[20] Dong W., Dong Z., Mao X., Sun Y., Li F., Shang D. (2016). Structure-activity analysis and biological studies of chensinin-1b analogues. Acta Biomater..

[21] Sun Y., Li H., Duan X., Ma X., Liu C., Shang D. (2024). Chensinin-1b Alleviates DSS-Induced Inflammatory Bowel Disease by Inducing Macrophage Switching from the M1 to the M2 Phenotype. Biomedicines.

[22] Li Z., Qu W., Zhang D., Sun Y., Shang D. (2023). The antimicrobial peptide chensinin-1b alleviates the inflammatory response by targeting the TLR4/NF-κB signaling pathway and inhibits Pseudomonas aeruginosa infection and LPS-mediated sepsis. Biomed. Pharmacother..

[23] Yang X.F., Hao Z.M., Cui X.Y., Liu W.Q., Li M.M., Shang D.J. (2025). Frog Skin Antimicrobial Peptide 3-13 and Its Analogs Alleviate Atherosclerosis Cholesterol Accumulation in Foam Cells via PPARγ Signaling Pathway. Cells.

[24] Anilkumar S., Wright-Jin E. (2024). NF-κB as an Inducible Regulator of Inflammation in the Central Nervous System. Cells.

[25] Kiernan R., Brès V., Ng R.W., Coudart M.P., El Messaoudi S., Sardet C., Jin D.Y., Emiliani S., Benkirane M. (2003). Post-activation turn-off of NF-kappa B-dependent transcription is regulated by acetylation of p65. J. Biol. Chem..

[26] Di Pietrantonio N., Di Tomo P., Mandatori D., Formoso G., Pandolfi A. (2023). Diabetes and Its Cardiovascular Complications: Potential Role of the Acetyltransferase p300. Cells.

[27] Ajoolabady A., Pratico D., Lin L., Mantzoros C.S., Bahijri S., Tuomilehto J., Ren J. (2024). Inflammation in atherosclerosis: Pathophysiology and mechanisms. Cell Death Dis..

[28] Bentzon J.F., Otsuka F., Virmani R., Falk E. (2014). Mechanisms of plaque formation and rupture. Circ. Res..

[29] Karunakaran D., Rayner K.J. (2016). Macrophage miRNAs in atherosclerosis. Biochim. Biophys. Acta.

[30] Dávalos A., Goedeke L., Smibert P., Ramírez C.M., Warrier N.P., Andreo U., Cirera-Salinas D., Rayner K., Suresh U., Pastor-Pareja J.C. (2011). miR-33a/b contribute to the regulation of fatty acid metabolism and insulin signaling. Proc. Natl. Acad. Sci. USA.

[31] Wang X., Feng L., Lu Y., Zhang H. (2024). miR-122/PPARβ axis is involved in hypoxic exercise and modulates fatty acid metabolism in skeletal muscle of obese rats. Heliyon.

[32] Nazari-Jahantigh M., Wei Y., Noels H., Akhtar S., Zhou Z., Koenen R.R., Heyll K., Gremse F., Kiessling F., Grommes J. (2012). MicroRNA-155 promotes atherosclerosis by repressing Bcl6 in macrophages. J. Clin. Investig..

[33] Hou J., Deng Q., Deng X., Zhong W., Liu S., Zhong Z. (2021). MicroRNA-146a-5p alleviates lipopolysaccharide-induced NLRP3 inflammasome injury and pro-inflammatory cytokine production via the regulation of TRAF6 and IRAK1 in human umbilical vein endothelial cells (HUVECs). Ann. Transl. Med..

[34] Barwal T.S., Singh N., Sharma U., Bazala S., Rani M., Behera A., Kumawat R.K., Kumar P., Uttam V., Khandelwal A. (2022). miR-590-5p: A double-edged sword in the oncogenesis process. Cancer Treat. Res. Commun..

[35] Yu M., Luo Y., Cong Z., Mu Y., Qiu Y., Zhong M. (2018). MicroRNA-590-5p Inhibits Intestinal Inflammation by Targeting YAP. J. Crohns Colitis.

[36] Guo Q., Su H., He J.B., Li H.Q., Sha J.J. (2018). MiR-590-5p alleviates intracerebral hemorrhage-induced brain injury through targeting Peli1 gene expression. Biochem. Biophys. Res. Commun..

[37] Xu L., Zhao G., Zhu H., Wang S., Sun A., Zou Y., Ge J. (2019). Peroxisome Proliferator-Activated Receptor-γ Antagonizes LOX-1-Mediated Endothelial Injury by Transcriptional Activation of miR-590-5p. PPAR Res..

[38] Dong W., Mao X., Guan Y., Kang Y., Shang D. (2017). Antimicrobial and anti-inflammatory activities of three chensinin-1 peptides containing mutation of glycine and histidine residues. Sci. Rep..

[39] Wu D., Fu L., Wen W., Dong N. (2022). The dual antimicrobial and immunomodulatory roles of host defense peptides and their applications in animal production. J. Anim. Sci. Biotechnol..

[40] Pantic J.M., Jovanovic I.P., Radosavljevic G.D., Arsenijevic N.N., Conlon J.M., Lukic M.L. (2017). The Potential of Frog Skin-Derived Peptides for Development into Therapeutically-Valuable Immunomodulatory Agents. Molecules.

[41] Apostolopoulos V., Bojarska J., Chai T.T., Elnagdy S., Kaczmarek K., Matsoukas J., New R., Parang K., Lopez O.P., Parhiz H. (2021). A Global Review on Short Peptides: Frontiers and Perspectives. Molecules.

[42] Järvinen T.A., May U., Prince S. (2015). Systemically Administered, Target Organ-Specific Therapies for Regenerative Medicine. Int. J. Mol. Sci..

[43] Carrascosa J.M. (2013). Immunogenicity in biologic therapy: Implications for dermatology. Actas Dermosifiliogr..

[44] Shang D., Meng X., Zhang D., Kou Z. (2017). Antibacterial activity of chensinin-1b, a peptide with a random coil conformation, against multiple-drug-resistant Pseudomonas aeruginosa. Biochem. Pharmacol..

