# Supplementary material for: The Frog Skin-Derived Antimicrobial Peptide Suppresses Atherosclerosis by Modulating the KLF12/p300 Axis Through miR-590-5p
Source: Int J Mol Sci. 2025 Nov 27;26(23):11497. doi: 10.3390/ijms262311497 (PMC12691761; doi:10.3390/ijms262311497)
Supplement: Supplementary file 1 [file ijms-26-11497-s001.zip › Supplementary Figure.pdf]

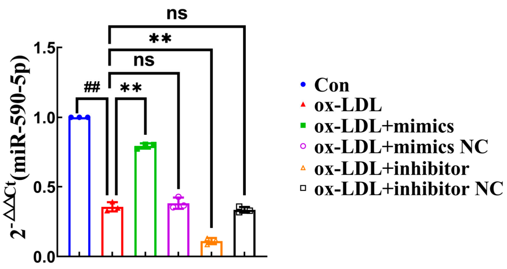

Supplementary Figure S1 RT-qPCR analysis of miR-590-5p expression in ox-LDL-induced foam cells following transfection with miR-590-5p mimics (50 nM, to overexpress miR-590-5p) or treatment with miR-590-5p inhibitor (50 nM, to knockdown miR-590-5p)(n = 3). One-way ANOVA was used to compare multiple groups for each mutation. Results are expressed as mean ± SEM from at least three independent biological replicates. Individual data points from all independent replicates are displayed in the graphs; ##p < 0.01 vs. the control group; ns: not significant, **p < 0.01 vs. the ox-LDL group.

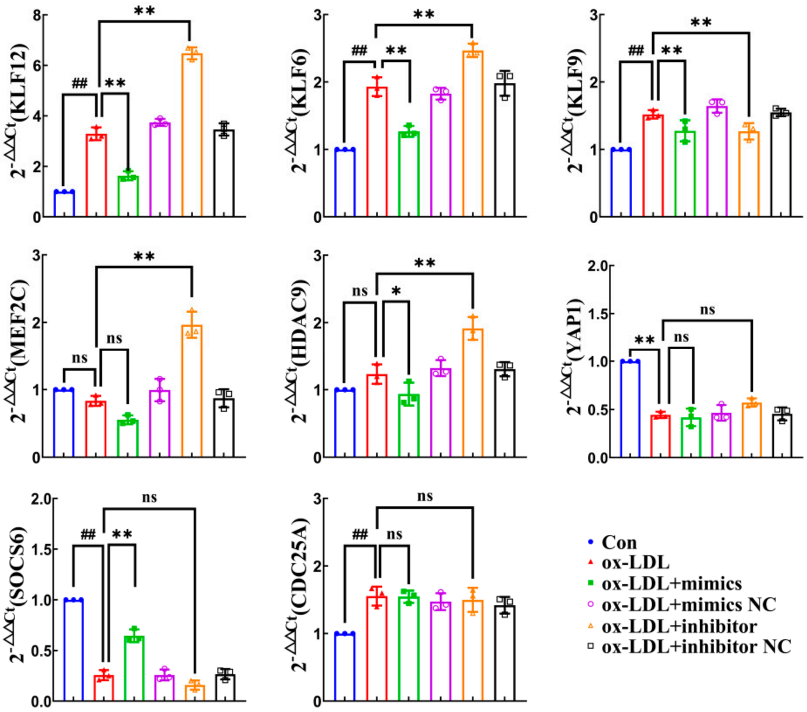

Supplementary Figure S2 RT-qPCR analysis of predicted miR-590-5p target genes in ox-LDL-induced foam cells. PMA-differentiated THP-1 macrophages were treated for 24 h under the following conditions: Con (untreated control); ox-LDL; ox-LDL + miR-590-5p mimics (50 nM); ox-LDL + mimics NC (50 nM, scrambled sequence); ox-LDL + miR-590-5p inhibitor (50 nM); and ox-LDL + inhibitor NC (50 nM, scrambled sequence) (n = 3). One-way ANOVA was used to compare multiple groups for each mutation. Results are expressed as mean ± SEM from at least three independent biological replicates. Individual data points from all independent replicates are displayed in the graphs; ns, not significant, ##p < 0.01 vs. the control group; ns, not significant, *p < 0.05 and **p < 0.01 vs. the ox-LDL group.

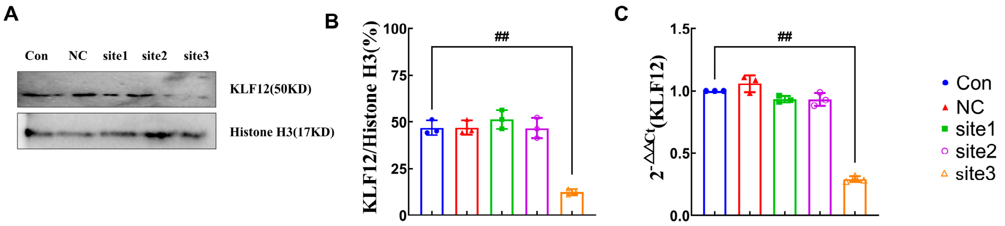

Supplementary Figure S3 (A&B) Western blot and quantitative analysis of KLF12 protein expression in ox-LDL-induced foam cells following treatment with three si-RNA to inhibit KLF12 expression($n = 3$). (C) RT-qPCR analysis of *KLF12* mRNA expression following treatment with three si-RNA(n = 3). All negative controls (NC) received a scrambled RNA sequence (50 nM). One-way ANOVA was used to compare multiple groups for each mutation. Results are expressed as mean ± SEM from at least three independent biological replicates. Individual data points from all independent replicates are displayed in the graphs; ##p < 0.01 vs. the control group.

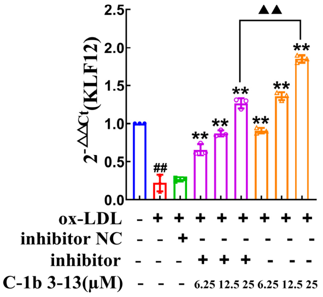

Supplementary Figure S4 RT-qPCR analysis of *KLF12* mRNA expression level in ox-LDL-induced foam cells following treatment: (**i**) AMP C-1b(3-13) alone (6.25, 12.5 or 25 μM), (**ii**) AMP C-1b(3-13) combined with miR-590-5p inhibitor (50 nM)(n = 3). One-way ANOVA was used to compare multiple groups for each mutation. Results are expressed as mean ± SEM from at least three independent biological replicates. Individual data points from all independent replicates are displayed in the graphs; ##p < 0.01 vs. the control group; **p < 0.01 vs. the ox-LDL group; and ▲▲ p < 0.01 vs. the C-1b(3-13) 25 μM group.
