# Supplementary material for: The Frog Skin-Derived Antimicrobial Peptide Suppresses Atherosclerosis by Modulating the KLF12/p300 Axis Through miR-590-5p
Source: Int J Mol Sci. 2025 Nov 27;26(23):11497. doi: 10.3390/ijms262311497 (PMC12691761; doi:10.3390/ijms262311497)
Supplement: Supplementary file 1 [file ijms-26-11497-s001.zip › Supplementary Table.pdf]

Table S1. Differential microRNAs

**ox-LDL vs Con**		**3-13 vs ox-LDL**	
hsa-miR-107	up	hsa-miR-101-3p	down
hsa-miR-12136	up	hsa-miR-126-3p	down
hsa-miR-124-5p	up	hsa-miR-1268b	up
hsa-miR-125b-1-3p	up	hsa-miR-127-3p	up
hsa-miR-126-3p	up	hsa-miR-1278	down
hsa-miR-127-3p	down	hsa-miR-142-3p	down
hsa-miR-132-3p	up	hsa-miR-142-5p	down
hsa-miR-132-5p	up	hsa-miR-143-5p	down
hsa-miR-142-3p	up	hsa-miR-15b-3p	down
hsa-miR-143-5p	up	hsa-miR-181a-3p	down
hsa-miR-15b-3p	up	hsa-miR-195-5p	down
hsa-miR-17-5p	up	hsa-miR-199a-3p<br>hsa-miR-199b-3p	down
hsa-miR-181a-3p	up	hsa-miR-200c-3p	down
hsa-miR-195-5p	up	hsa-miR-203a-3p	down
hsa-miR-199a-3p<br>hsa-miR-199b-3p	up	hsa-miR-205-5p	down
hsa-miR-203a-3p	up	hsa-miR-210-3p	down
hsa-miR-205-5p	up	hsa-miR-370-3p	down
hsa-miR-210-3p	up	hsa-miR-375-3p	down
hsa-miR-212-3p	up	hsa-miR-381-3p	down
hsa-miR-224-5p	up	hsa-miR-450a-5p	down
hsa-miR-320c	up	hsa-miR-452-5p	down
hsa-miR-33b-3p	up	hsa-miR-548u	down
hsa-miR-340-3p	up	hsa-miR-590-5p	up
hsa-miR-381-3p	up		
hsa-miR-4485-3p	up		
hsa-miR-516a-5p	up		
hsa-miR-548ad	up		
hsa-miR-548ay-5p	up		
hsa-miR-590-5p	down		
hsa-miR-6506-5p	up		
hsa-miR-654-3p	up		

Table S2. Target gene of miR-590-5p

**Target gene of miR-590-5p**		
YOD1	MAPK10	SON
ABCA1	MEF2C	SOS2
ARHGEF12	MIA3	SOWAHC
ARL1	NAA50	SPRY1
C1orf216	NHS	STAT3
CDC25A	NPAS3	SUZ12
CFL2	PANK3	TAGAP
CHD7	PCGF3	TBL1XR1
CHIC1	PCSK6	TEAD1
CNOT6	PDLIM5	TMEM59
CUX1	PDZD2	TNKS
DCAF7	PFKM	TNPO1
DIPK2A	PGM2L1	TRPM7
DST	PIK3R1	TWF1
DSTN	PIKFYVE	UBE2D3
EIF4EBP2	PLAG1	UBN2
FAM126B	PLEKHA1	YAP1
FOXK2	PM20D2	YOD1
GAB1	POLE3	ZBTB18
GPATCH2L	PRKCE	ZCCHC3
HDAC9	PRRC1	ZNF367
HNRNPU	PTPN14	ZNF592
IL6R	PURG	
ITPRIPL2	RAB22A	
KLF12	RTN4	
KLF6	SATB1	
KLF9	SC5D	
LSM3	SLMAP	
MAP3K8	SOCS6	

Table S3. Sequences of oligonucleotides

oligonucleotides	sequences
miR-590-5p mimics	sense strand: 5′-GAGCUUAUUCAUAAAAGUGCAG-3′<br>antisense strand: 5′-GCACUUUUAUGAAUAAGCUCUU-3′
miR-590-5p inhibitor	5′-CUGCACUUUUAUGAAUAAGCUC-3′
mimics negative control	sense strand: 5′-UUGUACUACACAAAAGUACUG-3′<br>antisense strand: 5′-GUACUUUUGUGUAGUACAAUU-3′
inhibitor negative control	5′-CAGUACUUUUGUGUAGUACAA-3′
si-KLF12 site1	sense strand: 5′-CGUCCACAACUAUCCCGAUAUTT-3′<br>antisense strand: 5′-AUAUCGGGAUAGUUGUGGACGTT-3′
si-KLF12 site2	sense strand:5′-CGAGGCAUUACCGCAAACAUATT-3′<br>antisense strand: 5′-UAUGUUUGCGGUAAUGCCUCGTT-3′
si-KLF12 site3	sense strand: 5′-GCGUUAAUGAAACUGGAUCUATT-3′<br>antisense strand: 5′-UAGAUCCAGUUUCAUUAACGCTT-3′
si-KLF12 negative control	sense strand: 5′-UUCUCCGAACGUGUCACGUTT-3′<br>antisense strand: 5′-ACGUGACACGUUCGGAGAATT-3′
